# Methylomic profiling of cortex samples from completed suicide cases implicates a role for *PSORS1C3* in major depression and suicide

**DOI:** 10.1038/tp.2016.249

**Published:** 2017-01-03

**Authors:** T M Murphy, B Crawford, E L Dempster, E Hannon, J Burrage, G Turecki, Z Kaminsky, J Mill

**Affiliations:** 1 University of Exeter Medical School, University of Exeter, Exeter, UK; 2 Department of Psychiatry, McGill University, Montreal, QC, Canada; 3 Department of Psychiatry, School of Medicine, Johns Hopkins University, Baltimore, MD, USA; 4 Department of Mental Health, Johns Hopkins Bloomberg School of Public Health, Baltimore, MD, USA; 5 King’s College London, MRC Social, Genetic and Developmental Psychiatry Centre, Institute of Psychiatry, Psychology and Neuroscience, London, UK

## Abstract

Major depressive disorder (MDD) represents a major social and economic health issue and constitutes a major risk factor for suicide. The molecular pathology of suicidal depression remains poorly understood, although it has been hypothesised that regulatory genomic processes are involved in the pathology of both MDD and suicidality. In this study, genome-wide patterns of DNA methylation were assessed in depressed suicide completers (*n*=20) and compared with non-psychiatric, sudden-death controls (*n*=20) using tissue from two cortical brain regions (Brodmann Area 11 (BA11) and Brodmann Area 25 (BA25)). Analyses focused on identifying differentially methylated regions (DMRs) associated with suicidal depression and epigenetic variation were explored in the context of polygenic risk scores for major depression and suicide. Weighted gene co-methylation network analysis was used to identify modules of co-methylated loci associated with depressed suicide completers and polygenic burden for MDD and suicide attempt. We identified a DMR upstream of the *PSORS1C3* gene, subsequently validated using bisulfite pyrosequencing and replicated in a second set of suicide samples, which is characterised by significant hypomethylation in both cortical brain regions in MDD suicide cases. We also identified discrete modules of co-methylated loci associated with polygenic risk burden for suicide attempt, but not major depression. Suicide-associated co-methylation modules were enriched among gene networks implicating biological processes relevant to depression and suicidality, including nervous system development and mitochondria function. Our data suggest that there are coordinated changes in DNA methylation associated with suicide that may offer novel insights into the molecular pathology associated with depressed suicide completers.

## Introduction

Major depressive disorder (MDD) represents a major social and economic health issue, affecting over 350 million people worldwide.^1^ MDD is associated with a reduced life expectancy and constitutes a major risk factor for suicide, a leading cause of mortality among young people in developed countries.^1^ Every year, almost one million people die from suicide globally with suicide attempts up to 20 times more frequent than completed suicide.^1^ The risk for suicidal acts is multifactorial, and consists of a range of biological, psychiatric, psychosocial and cultural risk factors.^2^ Currently, the molecular pathology of MDD leading to suicide remains poorly understood.

To date, large-scale genome-wide association studies of MDD have, with the exception of two loci associated with MDD in a large cohort of Chinese women,^3^ failed to identify robust replicated genetic loci.^3, 4^ These results indicate that the risk of MDD is unlikely to be increased by the effect of one or a few single-nucleotide polymorphisms but instead is highly polygenic in nature.^3^ Despite advances in understanding the genetic epidemiology of MDD, little is known about the mechanisms by which MDD risk variants mediate disease susceptibility in the brain.^5, 6^ Recent studies have started to examine the role of epigenetic processes—acting to regulate gene expression via modifications to DNA, histone proteins and chromatin—in a number of complex disease phenotypes.^7^ The epigenome is potentially malleable—changing with age and in response to specific environmental and psychosocial factors^8^—providing a mechanism for the interaction between genotype and the environment.^9^ Epigenetic processes, including DNA methylation, have recently been implicated in the aetiology of numerous mental health disorders,^10, 11, 12, 13, 14^ including MDD^15, 16, 17^ and suicidal behaviour.^18, 19, 20, 21^

To date, studies examining DNA methylation differences associated with MDD have primarily used peripheral tissue^15, 16, 17^ and few have examined DNA methylation changes in the brains of depressed individuals^22, 23, 24^ and depressed suicide completers.^22, 25^ Here, we describe a study of MDD suicide-associated methylation variation in matched cortical brain samples and its relationship to MDD and suicide attempt polygenic risk scores (PRS's). We profiled DNA methylation in two cortical brain regions (Brodmann Area 11 (BA11) and Brodmann Area 25 (BA25)) obtained from matched depressed suicide completers (*n*=20) compared with non-psychiatric, sudden-death controls (*n*=20). We identify evidence for altered DNA methylation in MDD suicide cases and MDD suicide-associated co-methylation modules that are associated with increased polygenic burden for suicide attempt.

## Materials and methods

### Samples

Tissue (*n*=75) from two regions of the cortex (BA11, *n*=40 and BA25, *n*=35) from 20 MDD suicide cases and 20 non-psychiatric sudden-death controls were obtained from the Douglas Bell Canada Brain Bank (http://douglasbrainbank.ca/; see Supplementary Table S1 for further details). Cases and controls did not differ significantly by ethnic background (*P*=0.748; Fisher’s exact test). BA25 is rostrally bound by the prefrontal BA11 region. Brain specimens used in this study were collected post-mortem following consent obtained from next-of-kin. The left hemisphere was kept frozen at −80 °C and cut into consecutive 1 cm-thick coronal sections that are then snap-frozen in liquid nitrogen and stored at −80 °C. Cases in this study were individuals who died by suicide as determined by the coroner and following psychological autopsies, which met the criteria for MDD as described elsewhere.^26, 27^ Controls were individuals who died suddenly in motor vehicle accidents or by cardiac arrest and did not have evidence of axis I disorders. Cases and controls were group-matched according to gender, age, post-mortem interval and tissue pH. The collection of brain tissue for banking by the Douglas Bell Canada Brain Bank is approved by the Douglas Mental Health University Institute’s Research Ethics Board (REB), and this study was approved by the University of Exeter Medical School REB. Genomic DNA was isolated from tissue samples using a standard phenol–chloroform extraction protocol and tested for purity and degradation using spectrophotometry and agarose gel electrophoresis.

### Methylomic profiling

Genomic DNA (500 ng) was treated in duplicate with sodium bisulfite using the EZ-96 DNA Methylation Kit (Zymo Research, Irvine, CA, USA) following the manufacturers’ standard protocol. DNA methylation was quantified using the Infinium HumanMethylation450 BeadChip Array (Illumina, San Diego, CA, USA) as previously described.^28^ Samples were randomised throughout all experimental procedures to avoid batch effects. The Genome Studio software (Illumina) was used to extract signal intensities for each probe and perform initial quality control. Further quality-control checks, quantile normalisation and separate background adjustment of methylated and unmethylated intensities of type I and II probes were employed using the wateRmelon package in R (available from the bioconductor www.bioconductor.org).^28^ Probes on the X- and Y-chromosomes were used to confirm sample sex. Comparison of 65 single-nucleotide polymorphism probes on the array confirmed that matched tissues were sourced from the same individual. All samples passed stringent quality-control measures (>1% of sites with a detection *P*-value>0.01). Probes with a detection *P*-value>0.01 in at least 1% of samples and/or a beadcount <3 in 5% of samples were removed across all samples. Nonspecific probes and probes on the X- and Y-chromosomes were removed.^29, 30^ The final analyses included 416 876 probes, and 75 samples (40 samples for brain region BA11 (20 MDD suicide cases and 20 non-psychiatric sudden-death controls) and 35 matched BA25 samples taken from the same individuals (17 MDD suicide cases and 18 non-psychiatric sudden-death controls)). Raw Illumina 450 K array data are deposited in the Gene Expression Omnibus database (accession number: GSE88890).

### Estimating differential brain cell counts

The R package (available at www.cran.r-project.org), Cell EpigenoType Specific mapper, designed for the quantification and normalisation of differing neuronal proportions in genome-wide DNA methylation data sets, was used to estimate brain cellular heterogeneity in all samples as previously described.^31^

### Data analyses of genome-wide DNA methylation

Statistical analyses were performed using R statistical package (version 3.2.1). The Beta value (*β*) is a ratio between methylated probe intensity and total probe intensities (sum of methylated and unmethylated probe intensities) and ranges from 0 to 1. Linear regression was used to examine differences in DNA methylation scores (reported as change in Beta value (Δ*β*)) between MDD suicide cases and controls at each CpG site, controlling for potential confounders (age, gender and estimated neuronal cell composition). Overlapping differentially methylated positions (DMPs) between brain regions (BA11 and BA25) was assessed by examining the correlation of effect sizes (mean adjusted beta difference) across the two brain regions of matched individuals using Pearson’s Correlation coefficient. We identified consistent MDD-associated DNA methylation differences across both brain regions by fitting a linear mixed-effect model using the lme4 R package (available at https://cran.r-project.org^32^). Where *β* (DNA methylation value) is the response variable, brain region and sample ID were included in the model as random effects and diagnosis, age, gender and cellular composition were included in the model as fixed effects.

### Region-based analysis

To identify differentially methylated regions (DMRs) in our data, we used the Python module *Comb-p*^33^ to group spatially correlated DMPs (seed *P*-value <1 × 10^−2^, minimum of three probes) at a maximum distance of 500 bp in each brain tissue. DMR *P*-values were corrected for multiple testing using Šidák correction.^34^

### Bisulfite pyrosequencing

We used bisulfite pyrosequencing to confirm MDD-associated differential DNA methylation across the *PSORS1C3*-associated DMR (spanning a region (chromosome 6:31148343–31148604 (hg19)), which encompasses 17 CpG sites (10 of which overlap with those measured by the 450 k array experiment)). A single amplicon (261 bp) was amplified using primers designed using the PyroMark Assay design software (Qiagen, Manchester, UK). Samples were bisulfite–polymerase chain reaction-amplified (HOT FIREPol DNA Polymerase, Solis Biodyne, Tartu, Estonia) using the primers and assay conditions in Supplementary Table S2 and sequenced using two sequencing primers to maximise coverage across the DMR using the Pyrosequencing Pyromark Q24 system (Qiagen). Correlation between the mean DNA methylation levels at 10 CpG sites quantified by bisulfite pyrosequencing and the Illumina 450k array was calculated using Pearson’s correlation coefficient. However, changes in DNA methylation across all 17 CpG sites between cases and controls across both brain regions were assessed by fitting a linear mixed-effect model by maximum likelihood using lme4 R package (as described above).

### Genotyping

Genomic DNA (200 ng) from each individual brain sample (*n*=40) was genotyped using the Illumina HumanOmniExpress BeadChip (Illumina). All samples were randomised with respect to gender and disease status to avoid batch effects. Illumina Genome Studio was used to call genotypes (using the HumanOmniExpress-12v1_C.egt cluster file) with the default GenCall cutoff of 0.15. Sex was confirmed by checking the reported sex of each individual against that predicted by the genetic data as described previously.^35^

### Polygenic risk scoring

PRS's were calculated using the *P*-values and log odds ratios from (i) the most recent Psychiatric Genetics Consortium genome-wide association study (GWAS) of MDD^4^ and (ii) a recent GWAS of suicide attempt.^36^ Risk scores were obtained following the method described by Purcell and colleagues^37^ using PLINK.^38^ For each individual, scores were generated from single-nucleotide polymorphisms selected by *P*-value-informed clumping (*P*-values<0.05) in PLINK,^38^ with a linkage disequilibrium cutoff of *r*^2^=0.1 within a 500-kb window. The numbers of single-nucleotide polymorphisms included in PRS for MDD, and suicide attempt calculation were 5267 and 3547, respectively.

### Weighted gene co-methylation network analysis

Network analysis was performed on residuals obtained after regressing out confounders (age, gender and estimated brain cell proportions) for each brain region (BA11 and BA25) separately using weighted gene co-methylation network analysis (WGCNA).^39^ For each brain region pairwise correlations were used to identify modules of highly co-methylated probes, independent of disease status as described previously.^11^ Briefly, an unsigned network was created using the *blockwiseModules* function based on a block size of 5000 and using a soft threshold parameter of 6. Each module was then labelled with a unique colour identifier and the module eigengene (ME) for each module (a weighted average methylation profile) was calculated based on the first principal component of the methylation matrix. To identify modules associated with diagnosis, a *t*-test was used to compare the mean ME values between cases and controls. Pearson’s correlation coefficients were used to examine association between ME values and continuous variables (MDD PRS and suicide attempt PRS). The module membership of each probe was calculated as the correlation between DNA methylation values and ME values. Probes with an absolute module membership value ⩾0.8 were used to identify the ‘hub’ genes in each module. Modules and their ‘hub’ genes were visualised using Cytoscape v3.2.^40^ A previously described logistic regression approach^41^ was used to test whether genes (Illumina UCSC gene annotation) annotated to probes in disease-associated modules predicted pathway membership while controlling for the number of probes annotated to each gene. Briefly, pathways were downloaded from the Gene Ontology (GO) website (http://geneontology.org/) and genes with at least one Illumina probe annotated to it and which mapped to at least one GO pathway were included. Pathways were filtered to those containing between 10 and 2000 genes, and a list of nominally significant pathways (*P*<0.05) was identified as described previously.^41^

### Replication analyses in an independent prefrontal cortex sample

Post-mortem prefrontal cortical tissue samples were obtained from the National Institute of Child Health and Human Development Brain and Tissue Bank for Developmental Disorders at the University of Maryland as described in detail elsewhere.^18^ These brain samples include suicide cases whose psychiatric diagnoses are unclear. DNA methylation data were available from sorted neuronal and non-neuronal cells obtained from 23 suicide cases and 33 non-suicide controls. Cell sorting was performed as described previously.^31^ DNA methylation was quantified using the Illumina Infinium HumanMethylation450 BeadChip array, with data normalisation and pre-processing performed using wateRmelon as described above. Brown’s method^42^ was used to calculate a combined *P*-value for the *PSORS1C3*-associated region in the suicide replication cohort.

## Results

### Brief overview of experimental approach

An overview of the methodological approach used in this study is given in Supplementary Figure 1. Briefly, we assessed genome-wide patterns of DNA methylation in two cortical brain regions (BA11 and BA25) obtained from depressed suicide completers (*n*=20) and matched non-psychiatric, sudden-death controls (*n*=20) using the Illumina 450 K HumanMethylation microarray (450 K array). Pre-processing, normalisation and stringent quality control were performed as previously described^28^ (see Materials and Methods for specific details). Our initial analyses focused on identifying DMPs and DMRs associated with MDD suicide cases compared with controls in both brain regions separately (Supplementary Figure 1A). We subsequently validated altered DNA methylation across a specific DMR using bisulfite pyrosequencing, and confirmed this association in an independent replication cohort (Supplementary Figure 1B). Next, PRS's were calculated for each sample using results from the most recent Psychiatric Genetics Consortium GWAS of MDD^4^ and a recent GWAS of suicide attempt^36^ (Supplementary Figure 1C). Finally, a system-level approach was used to identify networks of co-methylated loci associated with depressed suicide completion, in addition to polygenic burden for MDD and suicide attempt (Supplementary Figure 1D).

### Suicide-associated DMPs in cortical regions BA11 and BA25

**Figure 1:**
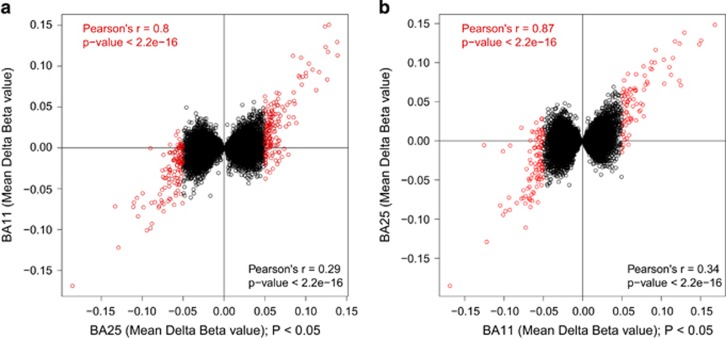
DNA methylation cross-tissue correlations. Correlation of effect sizes (mean adjusted beta difference) across the two brain regions of matched individuals at (**a**) nominally significant (*P*<0.05) BA25-associated DMPs and (**b**) nominally significant (*P*<0.05) BA11-associated DMPs. The mean adjusted beta differences at these loci were significantly positively correlated between brain regions (*P*<2.2 × 10^−16^). In addition, nominally significant DMPs exhibiting larger absolute beta differences (absolute Δ*β*⩾0.05; BA11 (*n*=181); BA25 (*n*=282) showed strong positive correlations when compared across the two brain regions (Pearson’s *r*=0.8, Pearson’s *r*=0.87, respectively, shown in red). BA11, Brodmann Area 11; BA25, Brodmann Area 25; DMP, differentially methylated position.

We assessed genome-wide patterns of DNA methylation in MDD suicide cases compared with non-psychiatric controls in two brain regions (BA11 and BA25) obtained from the same individuals using linear regression, controlling for age, gender and estimated neuronal proportion (see Materials and Methods). No DMP reached experiment-wide significance (*P*<1.66 × 10^−7^, threshold estimated from permutation analysis in a large data set (*n*=675 individuals) generated as part of an on-going study in our group);^43^ several showed suggestive evidence for association (*P*<5 × 10^−5^). The 10 most significantly differentially methylated loci identified in the MDD suicide group for brain regions BA11 and BA25 are listed in Supplementary Table S3 and include probes in the vicinity of a number of loci previously implicated in neuropsychiatric phenotypes. Next, we examined correlation of effect sizes (mean adjusted beta difference) across the two brain regions of matched individuals at (i) nominally significant (*P*<0.05) BA25-associated DMPs (Figure 1a) and (ii) nominally significant (*P*<0.05) BA11-associated DMPs (Figure 1b). The mean adjusted beta differences at these loci were significantly positively correlated between brain regions (BA25 versus BA11: Pearson’s *r*=0.29, *P*<2.2 × 10^−16^; BA11 versus BA25: Pearson’s *r*=0.34, *P*<2.2 × 10^−16^), with even stronger correlations across brain regions for DMPs showing large MDD suicide-associated differences (Δ*β*⩾0.05; BA25 versus BA11: Pearson’s *r*=0.8 (*P*<2.2 × 10^−16^), BA11 versus BA25: Pearson’s *r*=0.87 (*P*<2.2 × 10^−16^), Figure 1).

### Region-based analysis of altered DNA methylation in MDD suicide completers

**Figure 2:**
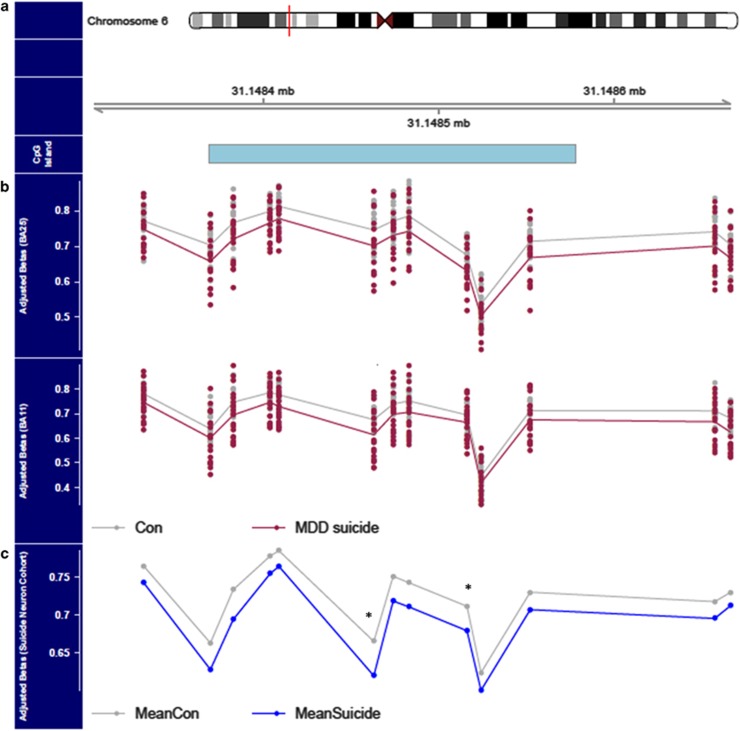
DNA hypomethylation of DMR upstream of the *PSORS1C3* non-coding gene. (**a**) Idiogram of chromosome 6 with genomic coordinates of DMR illustrated. The DMR—spanning 13 CpG sites—overlaps a CpG island (shown in blue). (**b**) *PSORS1C3*-associated DMR is hypomethylated across all 13 CpG sites in MDD suicide cases compared with controls in both brain regions. (**c**) Consistent with our findings, we observed DNA hypomethylation across all CpG sites present in the DMR in suicide cases compared with non-suicide controls in an independent suicide brain replication cohort. Two CpG sites (*) within the DMR were significantly differentially methylated between cases and controls (cg26818629 (*P*=0.03); cg26668675 (*P*=0.04)). DMR, differentially methylated region; MDD, major depressive disorder.

We used *Comb-p*^33^ to identify DMRs in MDD suicide cases compared with controls. Our analysis identified two significant (Sidak-corrected *P*-value<0.05) DMRs in BA25 (Table 1); a DMR on chromosome 13 in the promoter region of *DNAJC15* (Sidak-corrected *P*-value=0.03) and a DMR overlapping exon 3 of the *SNAI3* gene on chromosome 16 (Sidak-corrected *P*-value=0.005). No significant DMRs were identified in BA11 after correcting for multiple testing. Next, a cross-tissue DMR analysis was performed to identify regions consistently associated with MDD in both brain regions, using *P*-values obtained from fitting a linear mixed-effect model across both brain regions. This analysis identified three significant (Sidak-corrected *P*-value*<*0.05) DMRs (Table 1); the top-ranked DMR (spanning 13 CpG sites) is located in the promoter region of the *PSORS1C3* non-coding gene (Sidak-corrected *P*-value=8.02 × 10^−6^, Figure 2). The *PSORS1C3-*associated DMR is hypomethylated across all CpG sites in MDD suicide cases compared with controls in both brain regions (Figure 2b). In addition, we identified a DMR located in the third intron of the *TAPBP* gene (Sidak-corrected *P*-value=7.82 × 10^−5^) and a DMR located upstream in the promoter region of the *ATP5G2* gene (Sidak-corrected *P*-value=0.02).

### Validation and replication of the PSORS1C3-associated DMR

**Figure 3:**
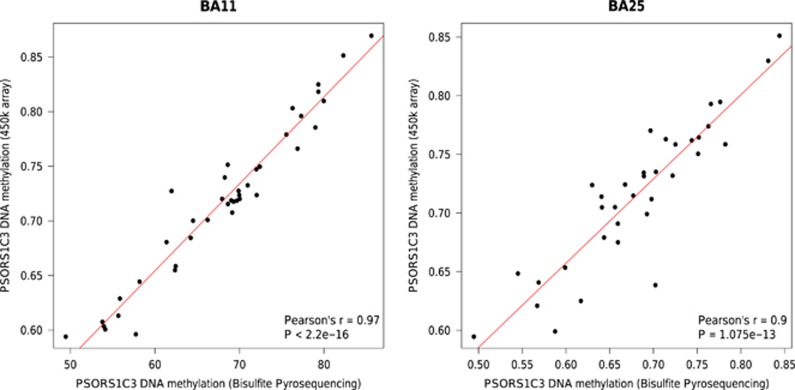
Technical validation of *PSORS1C3*-associated DMR using bisulfite pyrosequencing. Correlation of the mean DNA methylation values (at 10 CpG sites) obtained from the 450 k array (*y* axis) and bisulfite pyrosequencing (*x* axis) for samples in the brain region (**a**) BA11 (Pearson’s *r*=0.97; *P*-value<2.2 × 10^−16^) and (**b**) BA25 (Pearson’s *r*=0.97; *P*-value=1.07 × 10^−13^). BA11, Brodmann Area 11; BA25, Brodmann Area 25; DMR, differentially methylated region.

We next sought to technically validate our Illumina 450K array data across the *PSORS1C3* DMR using bisulfite pyrosequencing. The mean DNA methylation estimated by bisulfite pyrosequencing of the *PSORS1C3*-associated DMR was found to be strongly correlated with the Illumina 450K array data (BA25: Pearson’s *r*=0.97 (*P*<2.2 × 10^−16^); BA11 Pearson’s *r*=0.9 (*P*<1.075 × 10^−13^; Figure 3), in both brain regions, and confirmed DNA hypomethylation across the region (*P*=0.029) in suicide cases compared with controls at this locus (see Supplementary Table S4). We next examined 450 k array DNA methylation measures across this region in an independent set of prefrontal cortex (PFC) neuronal-sorted suicide brain samples (suicide cases (*n*=23) and non-suicide controls (*n*=33)). Consistent with our findings, we observed reduced DNA methylation across all CpG sites present in the DMR in suicide cases compared with non-suicide controls (Figure 2c). Although overall patterns of DNA methylation did not reach statistical significance (Brown’s *P*-value= 0.13), two CpG sites within the DMR were significantly differentially methylated between cases and controls (cg26818629 (*P*=0.03); cg26668675 (*P*=0.04), see Figure 2c; Supplementary Table S5). In contrast, there were no differences in DNA methylation at this region in DNA from non-neuronal-sorted cells obtained from the same individuals (*P*>0.1 for all sites; Supplementary Table S5), indicating that the effect was neuron-specific.

### Modules of co-methylated loci are associated with MDD suicide completers and suicide attempt PRS in brain region BA25

**Figure 4:**
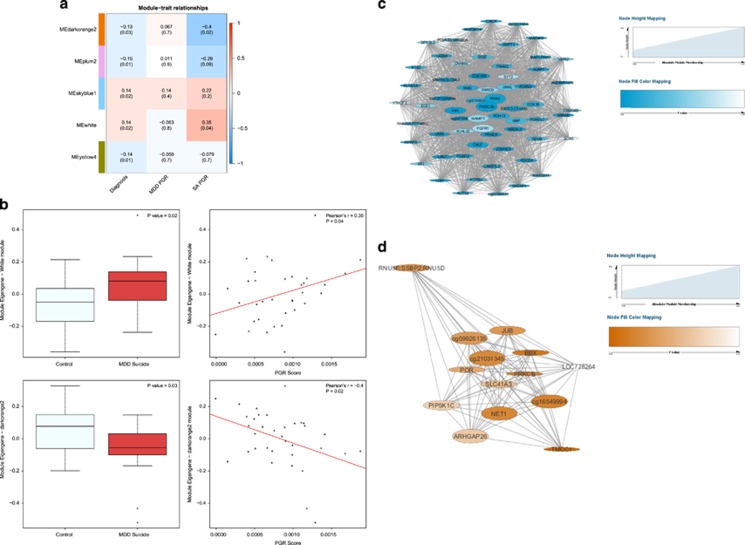
Modules of co-methylated loci are associated with both MDD suicide completers and SA PRS in brain region BA25. (**a**) Heatmap representing correlations between ME of diagnosis-associated modules and phenotype traits (diagnosis, MDD PRS and SA PRS). Each row represents a BA25 module, which is labelled with an arbitrary colour. Dark red indicates strong positive correlation, dark blue indicates strong negative correlation and white indicates no correlation (see colour scale bar). Uncorrected *P*-values are given in parentheses. The darkorange2 (*P*=0.02) and white (*P*=0.04) modules are also significantly associated with SA PRS in the BA25 brain region. (**b**) Boxplots and scatterplots of ME against diagnosis (control versus MDD suicide) and SA PRS, respectively. (**c**) Co-methylation networks between hub loci (module membership⩾0.8) in the white module, where larger circles indicate higher connectivity. Dark blue colour indicates a smaller *P*-value obtained from the probewise regression analysis examining the relationship between probes/genes and diagnosis (MDD suicide cases compared with controls), whereas white indicates a larger *P*-value. (**d**) Co-methylation networks between hub loci in the darkorange2 module, where larger circles indicate higher connectivity. Dark orange colour indicates a smaller *P*-value obtained from the probewise regression analysis examining the relationship between probes/genes and diagnosis (MDD suicide cases compared with controls), whereas white indicates a larger *P*-value. BA25, Brodmann Area 25; MDD, major depressive disorder; ME, module eigengene; PRS, polygenic risk score; SA, suicide attempt.

Two PRS for major depression and suicide attempt were generated from the most recent Psychiatric Genetics Consortium GWAS meta-analysis of MDD^4^ and a recent GWAS of suicide attempt,^36^ respectively. PRS scores for both MDD (*P*=0.01) and suicide attempt (*P*=0.07) were higher in MDD suicide cases compared with controls (Supplementary Figure 2), highlighting the utility of these scores. We next employed WGCNA^39^ to undertake a systems-level approach to identify networks of co-methylated modules associated with depressed suicide completers and MDD and suicide attempt PRS. WGCNA identified 80 modules of co-methylated probes in BA25 and 57 modules of co-methylated probes in BA11. The ME was used to assess the association between DNA methylation modules and phenotype traits of interest (i.e., diagnosis, suicide attempt PRS score and MDD PRS score). Five modules were found to be nominally significantly (*P*<0.05) correlated with diagnosis in brain region BA25 (Figure 4a), whereas only one module was associated with diagnosis in brain region BA11 (mean difference in ME between cases and controls (ΔME)=0.11, *P*=0.038). Interestingly, two MDD suicide-associated BA25 modules (the ‘white’ module (*n*=5581 probes): ΔME=0.14, *P*=0.02 and ‘darkorange2’ module (*n*=1980 probes): ΔME=−0.13, *P*=0.03) were also associated with a PRS score for suicide attempt (‘white’ module: *r*=0.35, *P*=0.04, ‘darkorange2’ module: *r*=−0.4, *P*=0.02 ) but not a PRS for MDD (Figure 4). Hub genes (absolute module membership⩾0.8) and their intramodule connectivity for the ‘white’ and ‘darkorange2’ modules are illustrated in Figure 4c and Figure 4d, respectively.

To facilitate biological interpretation of these MDD suicide-associated modules, we performed gene ontology enrichment analysis on genes annotated to probes in both modules using a logistic regression method previously described.^41^ The ‘darkorange2’ module was highly enriched for biological processes related to homophilic cell adhesion (GO:0007156, *P*-value=2.33 × 10^−9^) and establishment of cell polarity (GO:0030010, *P*-value=8.13 × 10^−6^). In contrast, the ‘white’ module was highly enriched for pathways related to nervous system development (GO:0007399, *P*-value=4.08 × 10^−6^), chromatin modification (GO:0016568, *P*-value=4.6 × 10^−6^) and mitochondria function (GO:0005739, *P*-value=6.04 × 10^−6^). A full list of all significantly (*P*-value<0.05) enriched pathways in the ‘darkorange2’ and ‘white’ modules are available in Supplementary TableS6 and TableS7, respectively. These analyses suggest that the coordinated changes in DNA methylation and suicide attempt PRS may contribute to disease pathology associated with depressed suicide completers.

## Discussion

In this study, we assessed genome-wide patterns of DNA methylation in DNA obtained from MDD suicide cases compared with non-psychiatric, sudden-death controls in two cortical brain regions (BA11 and BA25) using the Illumina 450 K array. To our knowledge, this represents the most extensive methylomic study of depressed suicide completers using post-mortem brain tissue to date.

We first examined site-specific genome-wide patterns of DNA methylation in MDD suicide cases compared with controls. Although no DMP reached experiment-wide significance, a number of DMPs identified have been previously implicated in neuropsychiatric phenotypes. For example, cg16292768 (intronic region of the *Clusterin* gene and upstream of *MIR6843*), which is hypomethylated in BA11 (*P*=3.35 × 10^−5^) of depressed suicide cases compared with controls, is associated with neuritic amyloid plaque burden, an early marker of Alzheimer’s pathology.^44^ Moreover, cg20434178, located in the promoter region of the autism-associated *DLX2* gene,^45, 46^ is hypermethylated in BA25 (*P*=5.4 × 10^−5^) in MDD suicide cases compared with controls. *DLX2* encodes a homeodomain-containing transcription factor and has been implicated in the generation of GABAergic cortical interneurons.^47^ A previous study examining DNA methylation changes in post-mortem PFC brain samples from MDD patients and controls identified DNA hypermethylation at the *PRIMA1* gene, and a concurrent decrease in gene expression.^24^ Interesting, cg23889730, a CpG site proximal to the *PRIMA1* gene region assessed in the MDD post-mortem brain study, was also hypermethylated in MDD suicide cases compared with controls (Δ*β*=0.02, *P*=0.04) in BA11 but not in BA25 in our study. Our data further support a role for *PRIMA1* in the pathogenesis of *MDD.*

To increase the power of our study to identify changes in DNA methylation between cases and controls, and given that DNA methylation at adjacent probes is often correlated, we employed the regional-based analysis, *comb-p,* to identify DMRs. Our analysis identified two DMRs in BA25: a DMR in a 5′ CpG island of the *DNAJC15* gene (Sidak-corrected *P*-value=1.7 × 10^−7^) and a DMR overlapping exon 3 of the *SNAI3* gene on chromosome 16 (Sidak-corrected *P*-value=1.7 × 10^−8^); *DNAJC15* is a mitochondrial protein that has an important role in the regulation of the mitochondrial function in macrophages and their response to inflammatory stimuli.^48^ Interestingly, CpG island methylation of the *DNAJC15* gene and concurrent transcriptional silencing have been previously reported,^48, 49^ suggesting that changes in DNA methylation at this locus are likely to alter gene expression levels. Recently, mitochondrial dysfunction has been hypothesised to have a role in the pathogenesis of MDD.^50^ *SNAI3* is a member of the *SNAIL* gene family, which have a role in mesodermal formation during embryogenesis^51^ but has not previously been linked to the pathogenesis of mental health disorders. In contrast to our finding in brain region BA25, no significant DMRs were identified in region BA11 from matched individuals. A cross-cortex DMR analysis identified three regions consistently associated with MDD suicide cases in both brain regions. The top-ranked DMR (spanning 13 CpG sites) is upstream of the *PSORS1C3* non-coding gene and overlaps a cluster of large intergenic non-coding RNAs associated with the *PSORS1C3* gene^52^ and is associated with a DNAse-hypersensitivity region.^53^ The *PSORS1C3-*associated DMR is hypomethylated across all CpG sites in MDD suicide cases compared with controls in both brain regions. We further validated this finding using bisulfite pyrosequencing in the same samples and confirmed reduced DNA methylation of the *PSORS1C3* DMR in an independent set of neuronal sorted post-mortem PFC samples from suicide cases compared with non-suicide control subjects. Although the function of the *PSORS1C3* gene product remains unknown, it is thought to potentially regulate nearby genes (for example, *POU5F1* and *HLA-C*), suggesting a prominent role in immune system regulation.^54^ Moreover, *PSORS1C3* is a known psoriasis susceptibility gene further supporting a role in immune system regulation.^54^ Of interest, we examined inter-individual variation at the *PSORS1C3*-associated DMR using a recently developed Blood Brain DNA Methylation Comparison Tool^55^ (data not shown). DNA methylation at this DMR is significantly correlated (*r*>0.43, *P*<0.0001 at each CpG site) between blood and multiple brain regions, suggesting that the DNA methylation patterns observed in the brain may also be detected in peripheral tissue.

Two additional MDD/suicide-associated DMRs were identified; a DMR located in the third intron of the *TAPBP* gene (Sidak-corrected *P*-value=7.82 × 10^−5^) and a DMR located upstream in the promoter region of the *ATP5G2* gene (Sidak-corrected *P*-value=0.02). The *TAPBP* gene, which is located within the major histocompatibility complex on chromosome 6, encodes a transmembrane glycoprotein that mediates interaction between major histocompatibility complex class I molecules and the transporter associated with antigen processing. In contrast, the *ATP5G2* gene encodes a subunit of mitochondrial ATP synthase.^56^ To the best of our knowledge, neither gene has been previously implicated in the pathology of MDD or suicidal behaviour. Taken together, our results provide evidence for a potential role for both immune regulation and mitochondria dysfunction in suicide and MDD. As DNA methylation can be influenced by specific genetic variants (for example, methylation quantitative trait loci (mQTLs)) in *cis* or *trans*, we examined all five DMRs identified in the regional analysis for evidence of the presence of mQTLs using our recently published data set examining mQTLs in the developing and adult human brain.^57^ We found no evidence of mQTLs in all DMR regions identified, suggesting that the changes in DNA methylation observed are not a result of genetic background.

In order to examine associations between DNA methylation and polygenic burden associated with both MDD and suicide, we generated two PRS's for MDD and suicide attempt, respectively, for each of the individuals profiled in this study. PRS scores for both MDD and suicide attempt were higher in MDD suicide cases compared with controls supporting the utility of these scores. Next, we used WGCNA to identify networks of co-methylated modules associated with depressed suicide completers and MDD and suicide attempt PRS. Our analysis identified two MDD suicide-associated BA25 modules (‘white’ module and ‘darkorange2’ module) that were also associated with a PRS score for suicide attempt but not a PRS for MDD. Furthermore, disease-associated co-methylation modules were enriched among gene networks, implicating biological processes relevant to depression and suicidality, including nervous system development and mitochondria function. These findings further support a hypothesis of mitochondria dysfunction in MDD and suicidal behaviour.

Despite the power of the methodological approaches used in this study, there are several limitations. First, our analysis utilised a small cohort of post-mortem brain samples (*n*=75) and is thus relatively underpowered to detect small changes in DNA methylation. Despite this we were able to identify a number of statistically significant DMRs. Moreover, we confirmed DNA hypomethylation of our top-ranked DMR by both bisulfite pyrosequencing and in post-mortem PFC samples obtained from an independent suicide cohort. Second, in this study we used bulk tissue from each brain region and cellular heterogeneity is a potentially important confounder in DNA methylation studies.^31, 58, 59^ However, we used a previously reported *in silico* method to estimate the neuronal proportion in each sample and included these estimates in our statistical models. Moreover, we examined DNA methylation changes in our top-ranked DMR in fluorescence-activated cell sorting-purified cells (neuronal and non-neuronal) in an independent PFC suicide replication cohort, thus allowing us to distinguish the methylation signature of the *PSORS1C3*-associated DMR in different brain cell types. Third, recent research has implicated the importance of other DNA modifications (i.e., 5-hydroxymethyl cytosine) in the brain.^60^ DNA methylation, as measured in this study, cannot be distinguished from 5-hydroxymethyl cytosine, and it is therefore plausible that the differences identified in this study are confounded by modifications other than DNA methylation. Future studies should attempt to examine the role of 5-hydroxymethyl cytosine in major depression and suicidal behaviour. Fourth, medication data were not available for all individuals; thus, we cannot rule out the possibility that the observed DNA methylation changes are confounded by medication. Finally, although our study presents evidence for novel DNA methylation changes associated with depressed suicide completers, further replication using a larger sample size is required to support these results. In addition, future studies could also examine the transcriptional consequences of the observed DNA methylation changes.

In summary, our data have identified a DMR, upstream of the *PSORS1C3* non-coding gene, which is consistently hypomethylated across both cortical brain regions in MDD suicide cases compared with controls and has identified MDD suicide-associated co-methylation modules that are specifically associated with suicide attempt PRS but not a MDD PRS.

## Figures and Tables

**Table 1:** Comb-p differentially methylated regional analysis

*Brain region*	*Hg19*	*Annotated gene (UCSC)*	*No. of probes*	*Slk* P*-value*	*Sidak* P*-value*
*BA25*					
	chr16:88744522–88744843	*SNAI3*	3	1.70E−08	0.005
	chr13:43597565–43598039	*DNAJC15*	4	1.71E−07	0.030
*Cross-tissue analysis (BA11 and BA25)*					
	chr6:31148332–31148667	*PSORS1C3*	13	3.14E−11	8.02E−06
	chr6:33280149–33280714	*TAPBP*	17	5.16E−10	7.82E−05
	chr12:54070931–54071195	*ATP5G2*	6	6.37−08	0.020

Abbreviations: BA11, Brodmann area 11; BA25, Brodmann area 25; Hg19, Human Genome version 19; UCSC, University of California, Santa Cruz Human Genome Browser. Stouffer–Liptak–Kechris correction (*slk*); one-step Šidák (1967) multiple-testing correction.
