# Supplementary figures and images for: Methylomic profiling of cortex samples from completed suicide cases implicates a role for PSORS1C3 in major depression and suicide
Source: Transl Psychiatry. 2017 Jan 3;7(1):e989–. doi: 10.1038/tp.2016.249 (PMC5545719; doi:10.1038/tp.2016.249)

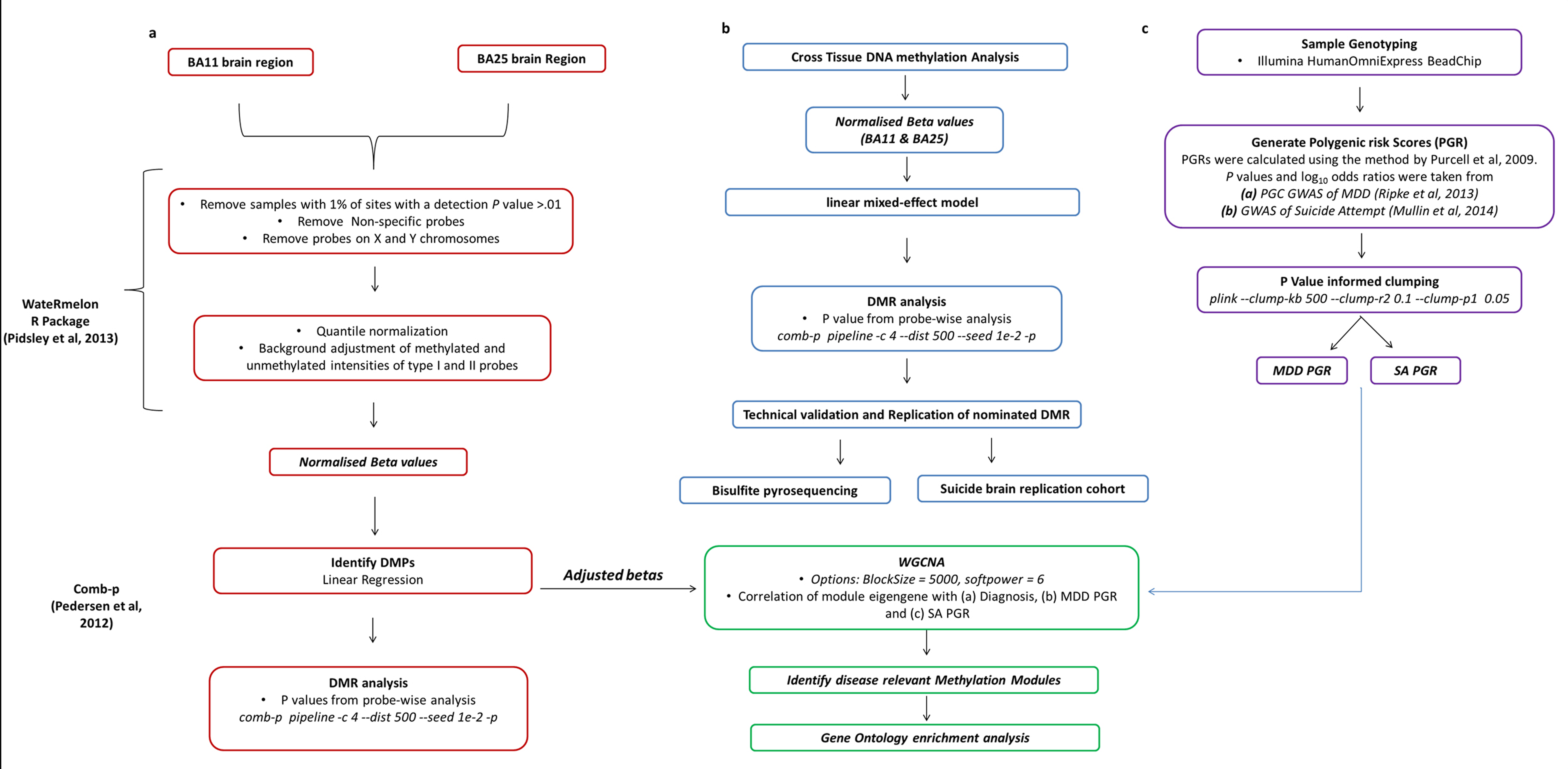

Supplement: Supplementary Figure 1 [file tp2016249x1.tif]

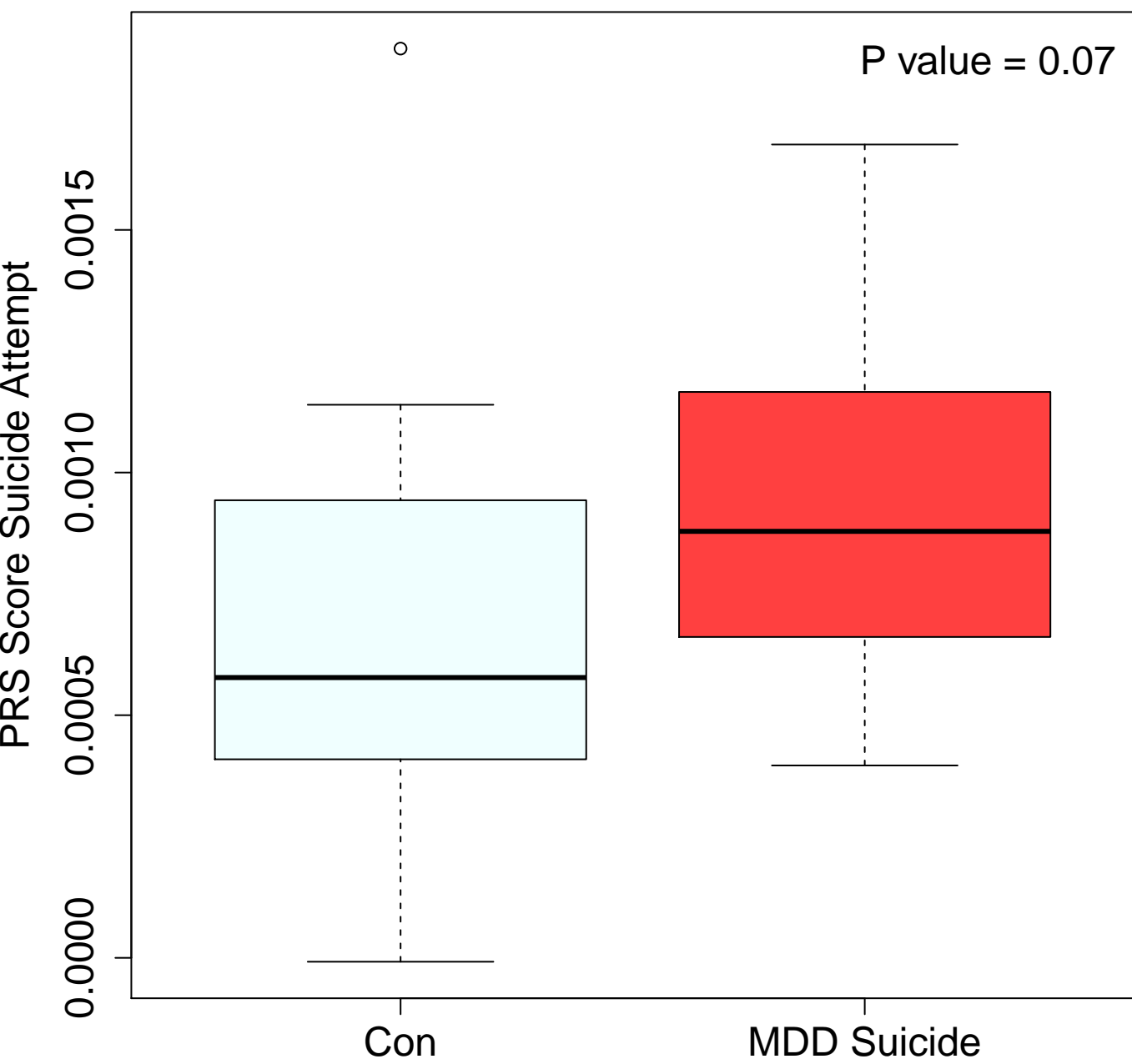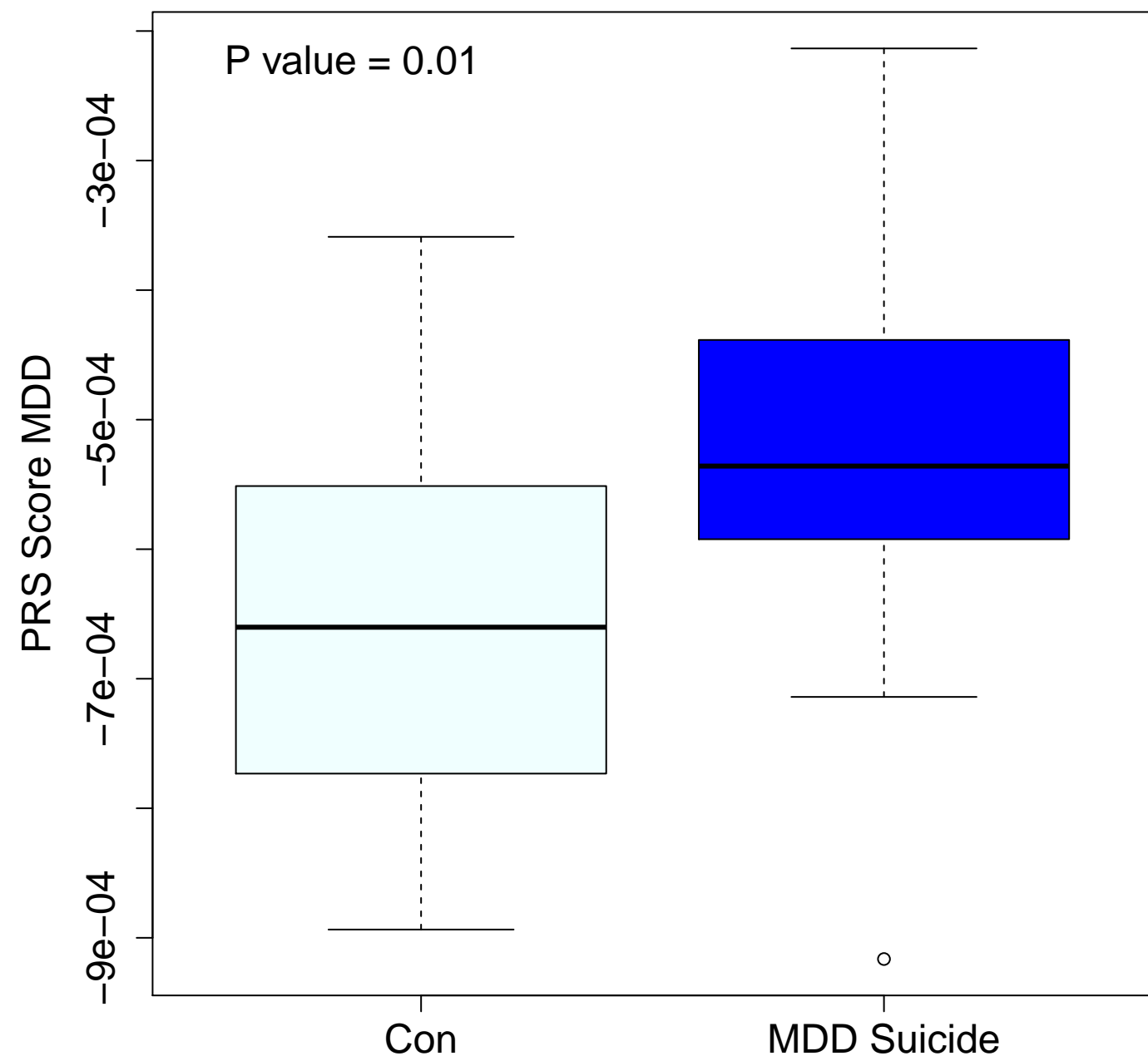

Supplement: Supplementary Figure 2 [file tp2016249x2.pdf]
