# Supplementary material for: Methylomic profiling of cortex samples from completed suicide cases implicates a role for PSORS1C3 in major depression and suicide
Source: Transl Psychiatry. 2017 Jan 3;7(1):e989–. doi: 10.1038/tp.2016.249 (PMC5545719; doi:10.1038/tp.2016.249)
Supplement: Supplementary Figure Legends [file tp2016249x3.docx]

**Supplemental Figure 1.** Flow chart describing the methodological approach used in this study.

**Supplemental Figure 2.** Boxplots comparing mean differences in suicide attempt (SA) (shown in red) and major depression disorder (MDD) (shown in blue) polygenic risk scores (PRS) between MDD suicide cases and controls.
