# Supplementary material for: Methylomic profiling of cortex samples from completed suicide cases implicates a role for PSORS1C3 in major depression and suicide
Source: Transl Psychiatry. 2017 Jan 3;7(1):e989–. doi: 10.1038/tp.2016.249 (PMC5545719; doi:10.1038/tp.2016.249)
Supplement: Supplementary Tables S1-S5 [file tp2016249x4.docx]

Supplementary Tables

| **Table S1. Characteristics of Discovery Cohort** | | | |  |  |  |  |  | |  |  |  |  |  |  |  |
| --- | --- | --- | --- | --- | --- | --- | --- | --- | --- | --- | --- | --- | --- | --- | --- | --- |
|  |  | **Age (Yrs)** | | | **Gender** | | **Post-mortem Interval (hrs)** | | |  |  |  |  |  |  |  |
|  | **N** | **Mean** | **SD** | | **M** | **F** | **Mean** | **SD** | |  |  |  |  |  |  |  |
| ***Brodmann Area 11*** |  |  |  | |  |  |  |  | |  |  |  |  |  |  |  |
| **MDD Suicide Cases** | 20 | 48.6 | 20.8 | | 5 | 15 | 20.0 | 15.6 | |  |  |  |  |  |  |  |
| **Controls** | 20 | 39.4 | 19.5 | | 4 | 16 | 26.1 | 20.5 | |  |  |  |  |  |  |  |
|  |  |  |  | |  |  |  |  | |  |  |  |  |  |  |  |
| ***Brodmann Area 25*** |  |  |  | |  |  |  |  | |  |  |  |  |  |  |  |
| **MDD Suicide Cases** | 17 | 49.5 | 22.4 | | 4 | 13 | 20.2 | 16.9 | |  |  |  |  |  |  |  |
| **Controls** | 18 | 41.2 | 19.6 | | 4 | 14 | 23.4 | 18.7 | |  |  |  |  |  |  |  |
| MDD: Major Depressive Disorder. Cases and controls did not differ significantly for age, gender or Post-mortem Interval (P > 0.05 for all comparisons). | | | | | | | | |  | |  |  |  |  |  |  |

| **Table S3. Top ten MDD suicide-associated DMPs in both brain regions** | | | | |  |  |  | |  |
| --- | --- | --- | --- | --- | --- | --- | --- | --- | --- |
| **Probe ID** | **Mean ∆β** | ***P* value** | **Hg19** | **Illumina annotation** | **Probe Type** | **Gene annotation from GREAT (Distance from TSS)** | | **SNP in Probe Sequence >10bp from SBE** | |
| *BA11* |  |  |  |  |  |  | |  | |
| cg26258213 | -0.078 | 8.00E-06 | Chr10:127220321 |  | I | *CTBP2 (-503869), C10orf137 (-187762)* | |  | |
| cg12734688 | -0.052 | 8.13E-06 | Chr1:48308390 |  | II | *LOC388630 (+154171), FOXD2 (+406702)* | |  | |
| cg03341758 | 0.016 | 1.09E-05 | Chr20:34330234 | *RBM39* | I | *RBM39 (+23)* | | rs2425124 | |
| cg14225665 | 0.029 | 1.32E-05 | Chr14:102394448 |  | II | *DYNC1H1 (-36416), PPP2R5C (+166314)* | |  | |
| cg17735716 | 0.020 | 1.43E-05 | Chr19:7692222 | *XAB2* | II | *C19orf79 (-2448), XAB2 (+2216)* | |  | |
| cg02508651 | 0.024 | 3.35E-05 | Chr17:46604554 |  | I | *SKAP1 (-96961), HOXB1 (+3717)* | |  | |
| cg16292768 | -0.042 | 3.35E-05 | Chr8:27467783 | *CLU* | II | *CLU (+4544), EPHX2 (+119139)* | |  | |
| cg07855967 | 0.016 | 4.00E-05 | Chr6:13486425 | *C6orf114;GFOD1* | I | *TBC1D7 (-157639), GFOD1 (+1443)* | |  | |
| cg01281851 | 0.061 | 4.65E-05 | Chr7:57271463 |  | II | *ZNF716 (-238419), ZNF479 (-63893)* | |  | |
| cg01310321 | 0.029 | 4.91E-05 | Chr10:134739934 |  | II | *NKX6-2 (-140398), TTC40 (+16154)* | |  | |
| *BA25* |  |  |  |  |  |  | |  | |
| cg10451253 | 0.027 | 9.49E-06 | Chr10:134739934 | *TMEM132E;C17orf102* | I | *TMEM132E (-62)* | |  | |
| cg21627409 | -0.045 | 2.29E-05 | Chr17:32907705 | *PCDHGA4;PCDHGA11* | I | *TAF7 (-109756), PCDHGC5 (-58701)* | |  | |
| cg19781637 | -0.074 | 2.69E-05 | Chr5:140810106 | *LHFPL2* | II | *LHFPL2 (+9324), SCAMP1 (+278985)* | |  | |
| cg10368447 | -0.019 | 3.32E-05 | Chr5:77935323 |  | I | *ESRP2 (-3824)* | |  | |
| cg19823793 | 0.027 | 3.37E-05 | Chr16:68273959 | *SGOL1* | I | *SGOL1 (+34)* | |  | |
| cg06967118 | -0.012 | 4.02E-05 | Chr3:20227663 | *FOXO3* | II | *FOXO3 (-2458)* | |  | |
| cg26666886 | 0.023 | 4.23E-05 | Chr6:108879610 | *ANKRD11* | II | *ANKRD11 (-1056)* | |  | |
| cg10082270 | -0.006 | 4.38E-05 | Chr16:89558024 | *FBXO31;MAP1LC3B* | I | *MAP1LC3B (-52)* | |  | |
| cg01785490 | -0.020 | 4.41E-05 | Chr16:87425748 |  | II | *AJAP1 (-55416), DFFB (+885844)* | |  | |
| cg20434178 | 0.038 | 5.41E-05 | Chr1:4659688 | *DLX2* | II | *DLX2 (-1207)* | |  | |
| MDD: Major depressive disorder. BA11: Brodman Area 11; BA25: Brodman Area 25, DMPs, differentially methylated positions, Adjusted *P* value; Bonferroni corrected, Hg19; Human Genome version 19, GREAT, Genomic Regions Enrichment of Annotations Tool,TSS, transcription start site. | | | | | | | | |  |
|  | | | | | |  |  | |  |

| **Table S4.** *PSORS1C3* DMR Pyrosequencing LMM results | | | | |
| --- | --- | --- | --- | --- |
| **CpG site** | **LMM coefficient** | **S.E** | **P value** | **Hg19** |
| cg17931227 | -5.326 | 2.206 | 0.021 | Chr6:31148370 |
| cg09179646 | -2.737 | 2.646 | 0.308 | Chr6:31148383 |
| cg22291762 | -4.285 | 2.408 | 0.084 | Chr6:31148404 |
| cg24427850 | -4.307 | 2.139 | 0.051 | Chr6:31148409 |
| CpG 5 | -3.042 | 2.298 | 0.194 | Chr6:31148414 |
| CpG 6 | -4.469 | 1.996 | 0.031 | Chr6:31148417 |
| CpG 7 | -6.299 | 2.393 | 0.012 | Chr6:31148457 |
| cg26668675 | -4.994 | 2.549 | 0.057 | Chr6:31148463 |
| CpG 9 | -7.123 | 2.574 | 0.009 | Chr6:31148467 |
| cg03078486 | -6.688 | 2.714 | 0.018 | Chr6:31148474 |
| cg22701603 | -5.480 | 2.686 | 0.048 | Chr6:31148483 |
| CpG 12 | -7.986 | 3.169 | 0.016 | Chr6:31148501 |
| CpG 13 | -5.164 | 2.371 | 0.036 | Chr6:31148513 |
| cg26818629 | -3.992 | 1.474 | 0.010 | Chr6:31148516 |
| cg27547543 | -4.640 | 2.005 | 0.026 | Chr6:31148524 |
| cg09357589 | -6.929 | 2.438 | 0.007 | Chr6:31148552 |
| CpG 17 | -7.436 | 3.197 | 0.025 | Chr6:31148571 |
| Mean | -5.373 | 2.375 | 0.029 |  |
| LMM Linear Mixed Effect Model; S.E. Standard Error; Hg19 Human Genome version 19. Mean is the LMM (controlling for confounders) of the average methylation score across all 17 CpG sites. | | | | |

| **Table S5. *PSORS1C3-*associated DMR analysis in replication cohort** | | | | |  |
| --- | --- | --- | --- | --- | --- |
|  | *Neuronal cells* | | *Non-Neuronal Cells* | | |
| **Probe ID** | **Regression coefficient** | **P value** | **Regression coefficient** | **P value** | |
| cg26818629 | -0.035 | **0.032** | -0.017 | 0.314 | |
| cg24427850 | -0.022 | 0.195 | -0.017 | 0.376 | |
| cg09357589 | -0.025 | 0.134 | -0.023 | 0.187 | |
| cg22291762 | -0.025 | 0.129 | -0.015 | 0.404 | |
| cg17931227 | -0.038 | 0.083 | -0.010 | 0.641 | |
| cg09179646 | -0.043 | 0.059 | -0.021 | 0.387 | |
| cg11811828 | -0.019 | 0.283 | -0.025 | 0.200 | |
| cg03078486 | -0.036 | 0.066 | -0.017 | 0.443 | |
| cg22701603 | -0.035 | 0.091 | -0.016 | 0.466 | |
| cg27547543 | -0.025 | 0.142 | -0.017 | 0.277 | |
| cg26668675 | -0.049 | **0.042** | -0.023 | 0.366 | |
| cg14036627 | -0.024 | 0.225 | -0.024 | 0.270 | |
| cg11805138 | -0.024 | 0.108 | -0.025 | 0.122 | |
| Linear regression (controlling for age and gender) was used to examine differences in DNA methylation at the *PSORS1C3*-associated DMR in an independent replication pre-frontal cortex suicide cohort. Significant (P < 0.05) are highlighted in bold. | | | | | |
|  |  |  |  |  |  |
|  |  |  |  |  |  |
